# Repousse spectaculaire d´une pelade décalvante traitée par triamcinolone en intramusculaire et stimulateur folliculaire topique

**DOI:** 10.11604/pamj.2021.40.106.31245

**Published:** 2021-10-15

**Authors:** Saer Diadie, Suzanne Oumou Niang

**Affiliations:** 1 Service de Dermatologie, Centre Hospitalier Universitaire Aristide Le Dantec, Dakar, Sénégal

**Keywords:** Alopecia areata, triamcinolone, follicular stimulator, regrowth, Pelade, triamcinolone, stimulateur folliculaire, repousse

## Abstract

We here report the case of a 42-year-old female patient presenting with alopecia areata that had progressed over the past month. The anamnesis revealed no psychoaffective irritative stimulus or atopy. Clinical examination was normal. The patient weighed 65kg with a BMI of 22kg/m^2^. Haemoglobin level was 12.5g/dL. Fasting blood glucose was 1.01g/L, TSHus and T4L were normal; 0.23 pmol/L and 13.32 pmol/L, respectively. Anti-nuclear and Anti-electroconvulsive Therapy (ECT) antibodies were negative. The diagnosis of isolated alopecia areata was retained. The patient was treated with intramuscular injections of 80mg of triamcinolone every ten days for 4 sessions (total dose of 320mg). Hair loss treatment topical spray was applied (12 sprays a day). Patients's outcome was marked by a spectacular regrowth of hair assessed at day 30 and day 60.

## Image en médecine

Il s´agissait d´une patiente âgée de 42 ans, reçue pour une pelade décalvante évoluant depuis 1 mois. L´anamnèse ne rapportait pas d´épine irritative psychoaffective ni d´atopie. L´examen clinique était normal. Elle pesait 65kg avec un IMC à 22kg/m^2^. Le taux d´hémoglobine était de 12,5g/dL. La glycémie à jeun était de 1,01g/L, le dosage du TSHus et T4libre était normal à 0,23 pmol/l et 13,32 pmol/L respectivement. La recherche des anticorps anti nucléaires et anti ECT était négative. Le diagnostic de pelade isolée retenu, la patiente a été traitée par triamcinolone, à raison d´une injection intramusculaire de 80 mg tous les dix jours pendant 4 séances soit un cumul de 320mg. Un traitement anti-chute topique était appliqué en 12 pulvérisations journalières. L´évolution était marquée par une repousse spectaculaire évaluée à J30 et J60.

**Figure 1:**
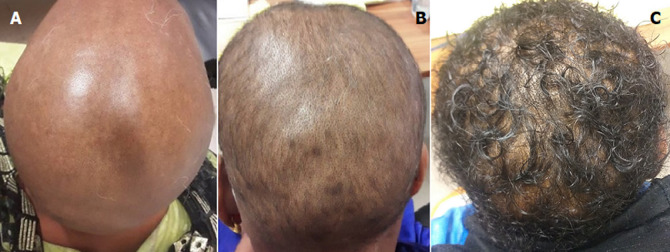
A) pelade décalvante J0; B) début de repousse à J30 de traitement (troisième injection intramusculaire de 80 mg de triamcinolone et application de stimulateur folliculaire); C) importante repousse à J60 avec un aspect touffu et lisse des cheveux (20 jours après la fin de la quatrième injection)

